# A Graphlet-Based Topological Characterization of the Resting-State Network in Healthy People

**DOI:** 10.3389/fnins.2021.665544

**Published:** 2021-04-28

**Authors:** Paolo Finotelli, Carlo Piccardi, Edie Miglio, Paolo Dulio

**Affiliations:** ^1^Department of Mathematics, Politecnico di Milano, Milan, Italy; ^2^Department of Electronics, Information and Bioengineering, Politecnico di Milano, Milan, Italy

**Keywords:** brain network, default mode network, fMRI, graphlet, network comparison, resting state

## Abstract

In this paper, we propose a graphlet-based topological algorithm for the investigation of the brain network at *resting state* (RS). To this aim, we model the brain as a graph, where (labeled) nodes correspond to specific cerebral areas and links are weighted connections determined by the intensity of the *functional magnetic resonance imaging* (fMRI). Then, we select a number of working *graphlets*, namely, connected and non-isomorphic induced subgraphs. We compute, for each labeled node, its *Graphlet Degree Vector* (GDV), which allows us to associate a GDV matrix to each one of the 133 subjects of the considered sample, reporting how many times each node of the atlas “touches” the independent orbits defined by the graphlet set. We focus on the 56 independent columns (i.e., non-redundant orbits) of the GDV matrices. By aggregating their count all over the 133 subjects and then by sorting each column independently, we obtain a *sorted node table*, whose top-level entries highlight the nodes (i.e., brain regions) most frequently touching each of the 56 independent graphlet orbits. Then, by pairwise comparing the columns of the sorted node table in the top-*k* entries for various values of *k*, we identify sets of nodes that are consistently involved with high frequency in the 56 independent graphlet orbits all over the 133 subjects. It turns out that these sets consist of labeled nodes directly belonging to the *default mode network* (DMN) or strongly interacting with it at the RS, indicating that graphlet analysis provides a viable tool for the topological characterization of such brain regions. We finally provide a validation of the graphlet approach by testing its power in catching network differences. To this aim, we encode in a *Graphlet Correlation Matrix* (GCM) the network information associated with each subject then construct a subject-to-subject *Graphlet Correlation Distance* (GCD) matrix based on the Euclidean distances between all possible pairs of GCM. The analysis of the clusters induced by the GCD matrix shows a clear separation of the subjects in two groups, whose relationship with the subject characteristics is investigated.

## Introduction

“Resting-state brain activity” is defined as the activity in the brain when a subject is awake but not performing a specific cognitive task or responding to external sensory stimuli.

The observation that *magnetic resonance imaging* (MRI)could be used to monitor temporally correlated low-frequency activity fluctuations in spatially remote brain areas led to widespread use of *resting-state functional magnetic resonance imaging* (rs-fMRI) to evaluate resting-state network properties (O’Connor and Zeffiro, 2019). Inside such a kind of network, the DMN represents the first and most studied resting-state subnetwork (Raichle et al., 2001; Greicius et al., 2003; Buckner et al., 2008), and it has been proposed to support “the construction of internal mental models based on mnemonic (limbic) systems” (Buckner et al., 2013). It is a complex large-scale network, crucial for understanding cognitive functions, that includes a number of highly interconnected brain regions. The DMN is of critical importance for maintaining the brain functions during the resting state. As the brain is engaged in goal-directed activity, the DMN experiences a progressive deactivation (see for instance; Anticevic et al., 2012). The main goal of this paper is to provide a topological characterization of the rs-fMRI network, i.e., the RS network generated by employing rs-fMRI data.

There is a general consensus on the fact that the areas forming the DMN are the posterior cingulate cortex (PCC), the precuneus (PCUN), the medial prefrontal cortex (mPFC), and the medial, lateral, and inferior parietal regions (mPL, LPL, IPL, resp.), which contribute to adaptive function, attention, and internal maintenance (Acheson and Hagoort, 2013). However, some authors (see for instance; Greicius et al., 2003; Buckner et al., 2008) also include in the anatomy of the DMN the ventral medial prefrontal cortex (vMPF), the inferior parietal lobule (IPL), the lateral temporal cortex (LTC), the dorsal-medial prefrontal cortex (dmPFC), and the hippocampal formation (HF). This limbic area is involved in the DMN in its entireness, including the hippocampus proper (HIP) as well as the entorhinal (EC) and parahippocampal (PHC) cortices. Differently, other authors (Andrews-Hanna et al., 2010, 2014), consider the DMN as a wider set of interconnected and anatomically defined brain regions. In particular, they speculate that the DMN consists of the following cerebral areas: The PCC, the PCUN, the mPFC, and the angular gyrus (AnG) should act as hubs, while the temporoparietal junction (TPJ), the lateral temporal cortex, and the anterior temporal pole constitute the dorsal medial system; and the hippocampus (HF+), the parahippocampus (PHC), the retrosplenial cortex (RSC), and the posterior inferior parietal lobe (pIPL) are the medial temporal subsystem. It follows that the DMN can be separated into hubs and subsections, which leads to the intriguing problem of providing a topological characterization of such brain regions.

In this paper, we use fMRI data obtained after parceling the brain into 94 cortical areas using the Harvard–Oxford Atlas (HOA) (see Table 1). Basically, brain parcellations divide the brain’s spatial domain into a set of non-overlapping regions characterized by some homogeneity with respect to the information provided by different image modalities, such as cytoarchitecture, anatomical connectivity, functional connectivity, or task-related activation. Hence, an atlas represents a certain labeling of brain structures. As a consequence, the nodes we are employing are labeled. From now on, we refer to “labeled node” simply as “node.” We wish to remark that other functional neuroimaging techniques can be employed, such as magnetoencephalography (MEG) (see for example; Finotelli et al., 2016) as well as different models of investigation, as shown in Finotelli and Dulio (2015).

**TABLE 1:** Harvard–Oxford Atlas (HOA) nodes and corresponding cerebral areas.

Node	Cerebral area	Node	Cerebral area
1	Left frontal pole	48	Right frontal pole
2	Left insular cortex	49	Right insular cortex
3	Left superior frontal gyrus	50	Right superior frontal gyrus
4	Left middle frontal gyrus	51	Right middle frontal gyrus
5	Left inferior frontal gyrus, pars triangularis	52	Right inferior frontal gyrus, pars triangularis
6	Left inferior frontal gyrus, pars opercularis	53	Right inferior frontal gyrus, pars opercularis
7	Left precentral gyrus	54	Right precentral gyrus
8	Left temporal pole	55	Right temporal pole
9	Left superior temporal gyrus, anterior division	56	Right superior temporal gyrus, anterior division
10	Left superior temporal gyrus, posterior division	57	Right superior temporal gyrus, posterior division
11	Left middle temporal gyrus, anterior division	58	Right middle temporal gyrus, anterior division
12	Left middle temporal gyrus, posterior division	59	Right middle temporal gyrus, posterior division
13	Left middle temporal gyrus, temporooccipital part	60	Right middle temporal gyrus, temporooccipital part
14	Left inferior temporal gyrus, anterior division	61	Right inferior temporal gyrus, anterior division
15	Left inferior temporal gyrus, posterior division	62	Right inferior temporal gyrus, posterior division
16	Left inferior temporal gyrus, temporooccipital part	63	Right inferior temporal gyrus, temporooccipital part
17	Left postcentral gyrus	64	Right postcentral gyrus
18	Left superior parietal lobule	65	Right superior parietal lobule
19	Left supramarginal gyrus, anterior division	66	Right supramarginal gyrus, anterior division
20	Left supramarginal gyrus, posterior division	67	Right supramarginal gyrus, posterior division
21	Left angular gyrus	68	Right angular gyrus
22	Left lateral occipital cortex, superior division	69	Right lateral occipital cortex, superior division
23	Left lateral occipital cortex, inferior division	70	Right lateral occipital cortex, inferior division
24	Left intracalcarine cortex	71	Right intracalcarine cortex
25	Left frontal medial cortex	72	Right frontal medial cortex
26	Left juxtapositional lobule cortex (formerly supplementary motor cortex)	73	Right juxtapositional lobule cortex (formerly supplementary motor cortex)
27	Left subcallosal cortex	74	Right subcallosal cortex
28	Left para cingulate gyrus	75	Right para cingulate gyrus
29	Left cingulate gyrus, anterior division	76	Right cingulate gyrus, anterior division
30	Left cingulate gyrus, posterior division	77	Right cingulate gyrus, posterior division
31	Left precuneus cortex	78	Right precuneus cortex
32	Left cuneal cortex	79	Right cuneal cortex
33	Left frontal orbital cortex	80	Right frontal orbital cortex
34	Left parahippocampal gyrus, anterior division	81	Right parahippocampal gyrus, anterior division
35	Left parahippocampal gyrus, posterior division	82	Right parahippocampal gyrus, posterior division
36	Left lingual gyrus	83	Right lingual gyrus
37	Left temporal fusiform cortex, anterior division	84	Right temporal fusiform cortex, anterior division
38	Left temporal fusiform cortex, posterior division	85	Right temporal fusiform cortex, posterior division
39	Left temporal occipital fusiform cortex	86	Right temporal occipital fusiform cortex
40	Left occipital fusiform gyrus	87	Right occipital fusiform gyrus
41	Left frontal operculum cortex	88	Right frontal operculum cortex
42	Left central opercular cortex	89	Right central opercular cortex
43	Left parietal operculum cortex	90	Right parietal operculum cortex
44	Left planum polare	91	Right Planum Polare
45	Left Heschl’s gyrus (includes H1 and H2)	92	Right Heschl’s gyrus (includes H1 and H2)
46	Left planum temporale	93	Right planum temporale
47	Left occipital pole	94	Right occipital pole

In agreement with (Finotelli et al., 2018), where an extended version of the DMN based on a graph theoretical and statistical analysis was provided, and with reference to Table 1, we consider the DMN defined by the left and right frontal poles (respectively, nodes 1 and 48); the left and right superior temporal gyrus, posterior division (respectively, nodes 10 and 57); the left and right middle temporal gyrus, posterior division (respectively, nodes 12 and 59); the left and right supramarginal gyrus, posterior division (respectively, nodes 20 and 67); the left and right angular gyrus (respectively, nodes 21 and 68); the left and right frontal medial cortices (respectively, nodes 25 and 72); the left and right cingulate gyrus, posterior division (respectively, nodes 30 and 77); and the left and right precuneus (respectively, nodes 31 and 78).

The main goal of this paper is to topologically explore the RS network and, as a consequence, the DMN and its principal connections with other cerebral areas. To this aim, we exploit *graphlets*, namely, connected non-isomorphic induced subgraphs of a network (Pržulj et al., 2004; Pržulj, 2007). We model the brain as a graph, where nodes correspond to specific cerebral areas and links are weighted connections determined by the intensity of the fMRI. Then, we select a number of working graphlets and we focus on the analysis of their *orbits*, i.e., nodes of the same graphlet that can be mutually interchanged under a graphlet automorphism. The frequency of appearance of the orbits is computed, for each node of the considered atlas, over the 133 graphs corresponding to the subjects. Sorting the resulting node list based on the frequency of occurrence of orbits, a *sorted node table* is obtained, whose columns are pairwise compared. This allows identifying sets of nodes that are consistently involved with high frequency in the graphlet orbits all over the 133 subjects. The analysis of such sets points out that the maximally recurrent orbits occur on a set of nodes that directly belong to the DMN or strongly interact with the DMN at the RS. We conclude that the graphlet approach is an effective tool to select a set of predominant regions at RS and thus a valid candidate for further topological brain network analyses.

In the term “graphlet,” the suffix “-let” recalls that the considered induced subgraphs are small with respect to the size of the network, pointing out the local nature of the approach. Focusing on small subgraphs could reveal important features of some special local neighborhoods where given networks are worth comparing. For instance, this approach has proved very successful in cellular network analysis, in particular in showing the exceptional high agreement of eukaryotic protein–protein interaction (PPI) networks with the geometric random graph model (Pržulj, 2007). In this paper, we aim to demonstrate that following a graphlet approach in the investigation of the brain network provides a nice topological interpretation of the brain functional connectivity.

In the second part of the paper, we give a validation of the graphlet method by testing its power in network comparison, by means of a network analysis based on a *graphlet correlation distance* (GCD) matrix, which defines the Euclidean distances between all possible pairs of subjects’ networks. The analysis of the clusters induced by the GCD matrix shows a clear and statistically significant separation in two groups, prompting future investigations on the predictive power of graphlets in brain network analysis.

The paper is organized as follows. First, we give some preliminaries and a detailed description of the employed material and methods (section “Materials and Methods”), including the algorithms we used. Then we present and discuss, in section “Results and Discussion,” the results obtained from the topological network analysis and from the network comparison. In section “Concluding Remarks,” we finally provide concluding remarks and future perspectives for this study.

## Materials and Methods

We model the brain network as an undirected graph, where nodes correspond to specific cerebral areas and links are weighted connections determined by the intensity of the fMRI, which constitutes a fundamental technique to examine brain activity by using blood oxygen level–dependent (BOLD) contrast.

It is well known that, even at RS (i.e., in absence of any task), the BOLD signal exhibits low-frequency spontaneous fluctuations, revealing how the brain preserves a kind of baseline activity though no external stimuli are received (see for instance; Snyder and Raichle, 2012 and the cited bibliography). The investigation of the RS condition leads to identification a number of brain regions generally referred to as the DMN. It is worth mentioning that the neuronal origin of such fluctuations was not immediately accepted, and the neuroscience community was mainly oriented to attribute them an artifactual nature related to the fMRI signals (see Glover and Lee, 1995; Friston et al., 1996; Wise et al., 2004). The neural origin was definitely established by showing that resting-state BOLD signals are temporally correlated within the somatomotor system (Biswal et al., 1995).

### Collecting the Functional Data

The dataset exploited in this paper is the same as in Finotelli et al. (2018, 2019). Each subject underwent a single MRI acquisition in a 1.5-T scanner (Siemens Magnetom Avanto, Germany) at Don Gnocchi Foundation, IRCCS Santa Maria Nascente (Milan, Italy). The following sequences have been collected: [A] rs-fMRI, BOLD EPI, collected at rest for approximately 6.6 min (TR/TE = 2,500/30 ms; resolution = 3.1 × 3.1 × 2.5 mm; matrix size = 64 × 64; number of axial slices = 39; number of volumes = 160). Subjects have been instructed to keep their eyes closed, to clear their mind from any specific thought, and not to fall asleep. [B] High-resolution T1-weighted 3D (TR/TE = 1,900/3.37 ms; resolution = 1 × 1 × 1 mm; matrix size = 192 × 256; number of axial slices = 176), as anatomical references for rs-fMRI analysis. [C] Conventional images (T2-weighted dual-echo turbo spin echo, FLAIR), to limit the risk of including subjects with concomitant vascular pathology or abnormal brain lesions. In particular, subjects with one or more macroscopic T2-weighted abnormalities located in the deep white matter (WM) or more than five abnormalities, maximum diameter < 5 mm, located in periventricular regions have been excluded from the study. All the rs-fMRI data have been preprocessed using the standard FSL processing pipeline (Smith et al., 2004; Jenkinson et al., 2012). All the details about the image preprocessing and the computation of anatomical and functional matrices are described in Finotelli et al. (2018, 2019).

### Managing the Functional Connectivity Matrices

BOLD signals are obtained by rs-fMRI methods; they are useful for investigating brain functional connectivity as well as for characterizing neurological diseases and mental disorders (Fox and Greicius, 2010). As detailed in Finotelli et al. (2018, 2019), we have analyzed the functional connectivity networks of 133 right-handed subjects, 51 males and 82 females of different ages, ranging from 6 to 79 years. For each subject, fMRI data have been parceled into 94 cortical areas using the Harvard–Oxford Atlas (HOA) (Table 1), obtaining 133 symmetric matrices of size 94 × 94 whose entries, ranging in the interval [−1,1], correspond to functional connectivity correlations. Such a 3D matrix of size 94 × 94 × 133 is denoted by *F*. With the entries of *F* being represented by correlation indices, we can separate positive and negative entries (i.e., positive and negative correlations of fMRI signal between pairs of nodes) and denote by *F*^+^ and *F*^−^ the resulting 94 × 94 × 133-sized 3D matrices, whose entries range in [0,1] and [−1,0], respectively. In this work, consistently with the majority of the literature on this topic, we only focus on the non-negative matrix *F*^+^, since the neurobiological description for positive correlation functional connectivity is more studied and detailed. As described in the following, to assess the robustness of the results, input data have been progressively refined by different thresholds. Additionally, the results have been compared with the outputs obtained from a randomized set of simulated data preserving the same size and node degree as the considered sample.

#### Ethical Approval

Every subject neither declared to take psychoactive medications at the time of the scan neither had a history of neurological or psychiatric disorders. According to the recommendations of the declaration of Helsinki for investigations on human subjects, the present study exploits methods previously approved by the Ethics Committee of Don Gnocchi Foundation (Milan, Italy). Written informed consent from all subjects to participate in the study was obtained before study initiation; for further details (see Finotelli et al., 2019).

### Graphlets

A powerful tool for investigating a number of network properties is represented by graphlets. First of all, let us provide a few useful definitions.

Let G = (V,E) be a graph, where V is the set of nodes, and E is a set of node pairs, i.e., a set of edges. The two nodes paired by an edge are said to be its endpoints. Let S be a subset of V: A graph H = (S, E’) is said to be an induced subgraph of G, if E’ consists of all the edges of E having both their endpoints in S.

**FIGURE 1:**
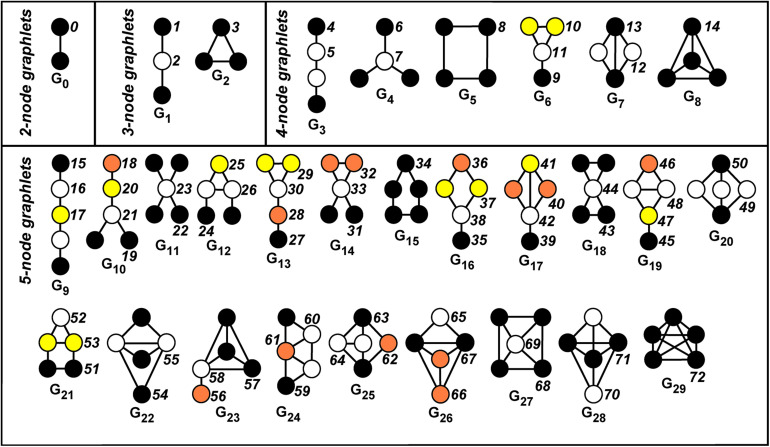
Automorphism orbits 0,1,2, …,72 for the 30 up to five-node graphlets *G*_0_,*G*_1_,…,*G*_29_. Nodes belonging to the same orbit of a graphlet have the same color. The non-redundant orbits we take into account are 0, 1, 2, 4, 6, 8, 9, 10, 11, 12, 13, 15, 18, 19, 22, 24, 25, 27, 29, 30, 31, 32, 33, 34, 35, 36, 37, 39, 40, 41, 42, 43, 45, 46, 48, 49, 50, 51, 52, 53, 54, 55, 56, 57, 58, 59, 60, 61, 62, 63, 64, 65, 66, 67, 68, 70.

A *graphlet* in G is a small connected induced subgraph of G (Pržulj et al., 2004; Pržulj, 2007). Figure 1 displays all the 30 possible graphlets, with up to five nodes, that can be extracted from a given graph. An *orbit* of a graphlet consists of different nodes that can be mutually interchanged under a graphlet automorphism. Hence, this relates to the symmetries of the graphlet.

More intuitively, the orbit defines the “topological character (or relevance)” of a node inside a graphlet, since it allows to distinguish between nodes of the graph G “touching” different nodes belonging to the graphlet *G*_*i*_ (in our case, *i* = 0,1,…,29, see Figure 1).

For example, let us consider the graphlet *G*_1_ in Figure 1. There exists an automorphism that exchanges the endpoints of the path, while no automorphism of the graphlet exists, which exchanges an endpoint with the middle vertex. This results in the existence of two orbits, called Orbit 1 and Orbit 2 (see again Figure 1, where different orbits inside a graphlet have different colors).

Hence, a node of the graph G can be described by a vector containing the counts of different kinds of graphlets which such a node “touches,” or equivalently, the topological characteristics (orbits) it plays within these graphlets.

As a first step, one selects a number of working graphlets having up to some prescribed number of nodes. We focus on up to five-node graphlets, namely, on the 30 graphlets *G*_0_,*G*_1_,…,*G*_29_ (see Figure 1), which globally define the 73 orbits *O*_0_,*O*_1_,…,*O*_72_. By leveraging the set of orbits, one can construct vectors collecting different measures associated with the given network. In detail, for each node, the corresponding *graphlet degree vector* (GDV) is formed by letting its *j*-th entry equal to the number of times the node “touches” (i.e., belongs to) the orbit *O_j*. The GDV can be computed by standard software packages, such as ORCA (Hocevar and Demsar, 2014). Many of the entries of the GDV have well-defined topological interpretations. For instance, the first entry is the degree of the associated node, the second entry is the number of induced paths of length two having the node as an endpoint, the third entry is the number of induced paths of lengths two having the node in the middle, and so on.

Some of the 73 orbits are dependent on other orbits previously considered, meaning that their count for any given node can be derived from the count of other orbits: The corresponding information is then redundant. In this case, the orbit can be neglected in the analysis. Focusing on up to five-node graphlets, it turns out that only 56 of the 73 orbits are non-redundant (see Yaveroğlu et al., 2014, Supplementary Material section). The list of the non-redundant orbits is reported in the caption of Figure 1.

### Frequency Table and Node Table

We consider the 133 square symmetric matrices $F_{h}^{+}$, *h* = 1,2,…,133, of size 94 × 94, corresponding to the positive correlations collected for all the involved subjects. Such matrices are then binarized and processed by ORCA (Hocevar and Demsar, 2014), to look for the graphlets up to five nodes and count, for each node, the occurrences of the corresponding 73 orbits.

Following Yaveroğlu et al. (2014), we define the GDVs by focusing only on the 56 non-redundant orbits listed in the caption of Figure 1. Actually, since the basic topological information of our interest is whether a given node *i* touches a given orbit *j*, rather than how many times, we binarize the entries of the GDVs to define binary graphlet degree vectors (BGDVs). Then, for each subject *h* = 1,2,…,133, we form a 94 × 56 matrix $M_{h}^{+}$ whose *i*-th row is the BGDV of node *i*. Finally, we sum up the 133 subject matrices $M_{h}^{+}$ to get a single 94 × 56-sized table $M_{f}=\sum_{h=1}^{133}M_{h}^{+}$, called *frequency table*. We remark that the entries (*i*,*j*) of *M_f*, called the *global orbit touching frequencies*, range in the interval [0,133]: They count how many subjects have a node *i* involved in orbit *j*.

Then we sort each column of *M_f* individually, so that the *i*-th position in each column is represented by the *i*-th highest global orbit touching frequency. The corresponding nodes are annotated in a separate 94 × 56 table. This provides two 94 × 56 sized tables, namely, the *sorted frequency table* (see Supplementary Table 1), which is the above-described matrix *M_f* with columns individually sorted, and the *sorted node table* (see Supplementary Table 2), where we take note of the node correspondence after sorting.

### Extracting Topologically Relevant Nodes

Our goal is to detect nodes that, all over the entire set of subjects, are characterized by high topological relevance in the RS network. We do this as follows.

**FIGURE 2:**
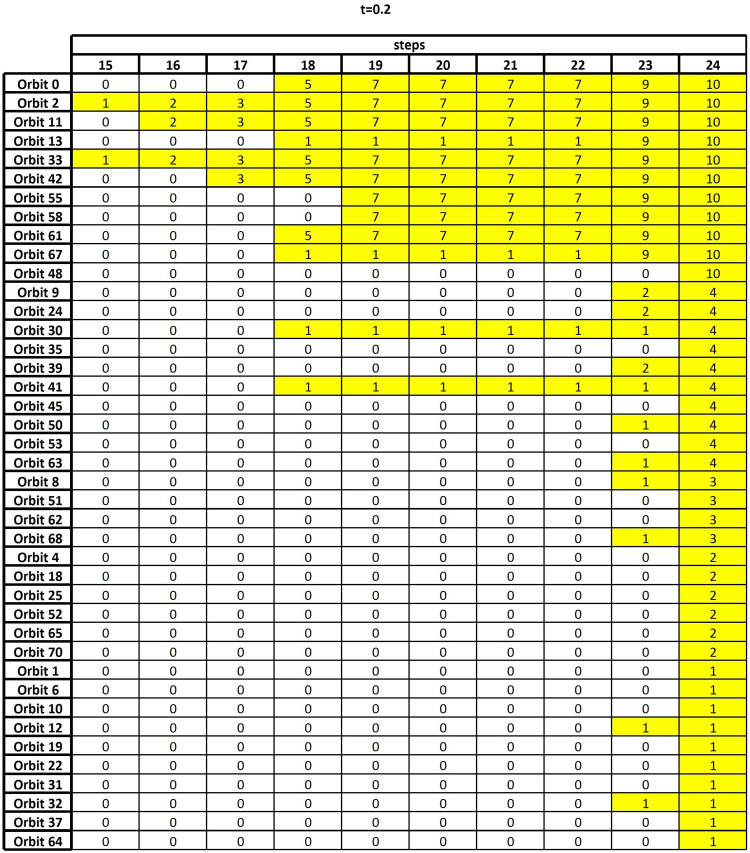
The output table, i.e., the result of the algorithm of section “Frequency Table and Node Table,” when the threshold *t* = 0.2 is considered.

(1) Sets of nodes are considered which are consistently involved, with high frequency, in a large number of orbits. To simplify the analysis, and to discard non-relevant information, we restrict our attention to the largest global orbit touching frequencies, specifically to the top quartile, which corresponds to consider only the first *r* = 24 rows of the sorted node table and of the sorted frequency table.
(2) *Column equivalence* is defined in terms of set equivalence, i.e., two columns of the sorted node table are equivalent if they contain the same set of nodes, regardless of the ordering.
(3) For each *k* = 1,2,…,*r*, we check column equivalence along the first *r*−*k* + 1 rows, for all pairs of columns of the sorted node table.
(4) For each *k*, and for each orbit *j* = 1,2,…,56, the orbits having column equivalence with *j* along the first *r*−*k* + 1 rows are detected.
(5) Results are summarized in an *output table*, namely a 56 × 24 sized table (see Figure 2) whose entry (*j*,*k*) is the number of columns equivalent to *j*, along the first *r*−*k* + 1 rows, in the sorted node table.

### Cluster Analysis

We further exploit the potentiality of graphlet analysis by comparing the different performance of the subjects at resting state through *graphlet correlation distance* (GCD), namely, the topological distance between pairs of networks defined in Yaveroğlu et al. (2014). GCD is defined as the Euclidean distance of the upper-triangle values of the two *graphlet correlation matrices* (GCM), as described here below.

First of all, starting from the above-introduced 94 × 56 GDV matrix $M_{h}^{+}$ associated with each subject *h* (*h* = 1,2,…,133), we form a 56 × 56 sized matrix $G\,C\,M_{h}^{+}$ whose entry (*l*,*m*) contains the Spearman correlation between columns *l* and *m* of $M_{h}^{+}$. Thus $G\,C\,M_{h}^{+}\,\left(l,m\right)$ quantifies how well the relationship between the columns *l* and *m* (*l*,*m* = 1,2,…,56) can be described by a monotonic function: In other words, it captures the tendency of the two columns to have the same increasing or decreasing behavior. This correlation is preferable to the usual linear Pearson correlation since the orbit counts are spread over different scales. Using matrices $G\,C\,M_{h}^{+}$, we then construct the symmetric 133 × 133 sized GCD matrix, which collects the Euclidean distances between all possible pairs of networks. Yaveroğlu et al. (2014) showed that GCD is clean of redundancies, encodes information about local network topology, and is a powerful comparing measure among networks in different domains such as computational biology and economics (Tantardini et al., 2019). Then, we perform a cluster analysis based on GCD. Among the several clustering techniques, we use agglomerative Hierarchical Cluster Analysis (Kaufman and Rousseeuw, 1990; Lior and Maimon, 2005) and, among the algorithms for defining the distance between clusters, we adopt Ward’s method (Ward, 1963; Thirion et al., 2014), since it is appropriate for Euclidean distances.

## Results and Discussion

In this section, we present the results obtained, as well as the corresponding discussion, by using the above-described graphlet methodology, first those concerning the GDV analysis and then those related to GCM comparison.

### GDV Analysis

As discussed in the previous section, the final result of the GDV analysis is an output table whose goal is to highlight the nodes which, all over the entire set of subjects, are characterized by high topological relevance. Figure 2 shows such an output corresponding to thresholding the functional connectivity matrix *F*^+^ at *t* = 0.2, i.e., only the entries whose weights are greater than or equal to 0.2 are retained, while the others are set to zero. In Supplementary Tables 3, 4, the analogous output tables for *t* = 0 and *t* = 0.1 are reported for comparison. We remark that changing the threshold implies changing the number and type of graphlets involving any given node, which, in turns, reflects in a change of the sorted node and frequency tables. This leads to possible fluctuations in the results, which are therefore compared over different threshold values to assure robustness (see below).

Figure 2 reports the nonzero part of the output table, namely, the 41 × 10 table obtained by removing from the output table, as defined in section “Frequency Table and Node Table,” the leftmost 14 columns and the bottom 15 rows, since all their entries are equal to zero. A zero entry means that no column equivalence occurs. Differently, a nonzero entry *x* in position (*j*,*k*) means that, at the *k*-th step of the algorithm, the orbit of row *j* shares the same nodes with other *x* orbits. For example, let us consider *k* = 19: It contains the result of checking set equivalence on the top 24−19 + 1=6 rows of the sorted node table. The entry *x* = 7 in the first row means that Orbit 0 contains the same six nodes as seven other orbits.

From the analysis of the output table and starting from the rows having the highest number of non-zero entries, we can easily reconstruct the process of orbit aggregation. Indeed, the two 1’s in the column *k* = 15 (the first column having non-zero entries) show that Orbit 2 and Orbit 33 are equivalent on the first 24−15 + 1=10 nodes. This means that these two orbits frequently interact with the same 10 nodes, not necessarily in the same order of occurrence. Of course, this also implies that the columns of the sorted node table corresponding to Orbit 2 and Orbit 33 will be equivalent in the remaining steps of the algorithm, since the requirement of column equivalence becomes less demanding. We resume this by saying that the equivalence between the columns corresponding to Orbit 2 and Orbit 33 persists since the 15-th step.

The three 2’s at the 16-th step show that Orbit 2 and Orbit 33 are further equivalent to Orbit 11, on the first nine nodes. At the 17-th step Orbit 42 also aggregates. At the 18-th step, Orbit 0 and Orbit 61 add to the previous list, thus forming a set *S* of 6 orbits whose corresponding columns of the sorted node table are equivalent on 24−18 + 1=7 nodes. At the same 18-th step, the four further 1’s indicate that the columns corresponding to Orbit 13, Orbit 30, Orbit 41, and Orbit 67 are pairwise equal in two different sets, which cannot be detected precisely at this stage.

At the 19-th step, the columns corresponding to Orbit 55 and Orbit 58 become equivalent to the previous six, so enlarging *S* to a set of 8 orbits, with persistent equivalence on six nodes. Concerning the pairwise aggregation of the columns corresponding to Orbit 13, Orbit 30, Orbit 41, and Orbit 67, no new information is obtained. The output remains stable from the 19-th to the 22-nd step, while at the 23-rd step, the two previous 1’s detected in the columns corresponding to Orbit 13 and Orbit 67 turn into 9, meaning that these two orbits add to the set *S*, so that we get 10 orbits that are equivalent on the first two nodes. Finally, at the last step, Orbit 48 is also included.

#### Interpreting the Results of the Topological Analysis

Investigating the topology of the orbits can unveil important neurobiological properties of the brain. Indeed, a connector hub may be thought of as a sorting center for information transmission among cerebral areas, while the fact that a node belongs to different cycles, or even to a clique, gives information on the resilience of the networks, i.e., on the plasticity of the brain.

The topological meaning of specific non-redundant orbits descends from their intrinsic structure. For instance (see also Pržulj, 2007; Yaveroğlu et al., 2014), Orbits 2, 11, 33, 42, 55, and 58 point out the attitude of a node to share information, since they are the center of the corresponding graph(let); hence, these orbits could characterize the existence of hubs. The connector hubs are believed to tune the connectivity of their neighbors in order to increase their modularity. In particular, it is speculated that such an action increases information integration across communities, global modularity, and cognitive performance (Bertolero et al., 2018). Differently, Orbits 65, 66, and 67 in graphlet *G*_26_ being part of different cycles could be associated with a kind of robustness of the brain network since information can be shared among the other nodes even when a connection should be removed from the network.

Let us now discuss the set of nodes emerging from the algorithm at the various steps. The main set is undoubtedly the one composed by six nodes and belonging to eight different equivalent orbits from the 19-th step on, with the further addition of three orbits in later steps. Such nodes are 1 (left frontal pole), 22 (left lateral occipital cortex, superior division), 31 (left precuneus cortex), 48 (right frontal pole), 69 (right lateral occipital cortex, superior division), and 78 (right precuneus cortex). Remarkably, nodes 1, 31, 48, and 78 all belong to the DMN and play a fundamental role (see, e.g., Mansouri et al., 2015; Utevsky et al., 2016; Zhan et al., 2017), while nodes 22 and 69 are of relevant neurobiological importance (see, e.g., Japee et al., 2015; de Schipper et al., 2018). The functional role of the above mentioned nodes is recalled in Table 2, while we refer the interested reader to Kolb and Whishaw (2009, 2014) for further details.

**TABLE 2:** Neurobiological relevant nodes emerging from the topological graphlet-based algorithm of section “Frequency Table and Node Table.”

Node	Name	Functional role
**1**	**Left frontal pole**	In human beings, the largest part of the PFC has the control of internal and purposeful mental action, also known as reasoning or prefrontal synthesis. The frontal cortex is the “action” cortex, much as the posterior cortex is the “sensory” cortex.
22	Left lateral occipital cortex, superior division	In Grill-Spector et al. (2001), the authors state that “the lateral occipital complex is a region of the brain that seems to play a central role in human object recognition.”
**31**	**Left precuneus cortex**	The precuneus is involved in memory tasks, with particular focus on spatial details. Together with the left prefrontal cortex it is involved in the recall of episodic memories including past episodes related to the self.
**48**	**Right frontal pole**	Liu et al. (2013) found that the right orbital frontal pole (FP) shows stronger connection probabilities to brain regions of the social emotion network (SEN), such as the orbito frontal cortex (OFC), amygdala, and temporal pole. The right lateral FP shows greater connection probabilities to the right dorso lateral prefrontal cortex, a critical node of the cognitive processing network (CPN). The right medial FP showed stronger connection probabilities to brain areas of the DMN, including the anterior cingulate cortex and medial prefrontal cortex.
69	Right lateral occipital cortex, superior division	The occipital lobe is one of the four major lobes of the cerebral cortex in the brain of mammals. The occipital lobe is the visual processing center of the mammalian brain containing most of the anatomical region of the visual cortex. The primary visual cortex is Brodmann area 17, commonly called V1 (visual one).
**78**	**Right precuneus cortex**	The precuneus is involved in several complex functions such as recollection and memory, integration of information (gestalt) relating to perception of the environment, cue reactivity, mental imagery strategies, episodic memory retrieval, and affective responses to pain.

*For each node, the anatomical name and functional role is provided. In black bold are denoted the nodes belonging to the DMN.*

Hence, by matching both the statistical outcomes obtained in Finotelli et al. (2018) and the outcomes in the above-cited literature, with the topological graphlet-based results of this study, we are led to propose that the RS network principally consists of the DMN and a few of the other brain areas that play a relevant topological role. Of course, we are aware that such a speculation requires precautions and must be supported by further analysis, such as the use of a different parceling.

#### Robustness With Respect to Different Threshold Levels

Let us now compare the three output tables shown in Supplementary Tables 3, 4 and Figure 2, obtained by thresholding the functional connectivity matrix *F*^+^ at *t=0*, *t* = 0.1, and *t* = 0.2, respectively. Focusing our attention on the orbits which sooner provide column equivalence, we realize that, regardless of the threshold value, they involve in all cases a subset consisting of the same six nodes. Indeed, from Supplementary Table 3 (*t=0*) the orbits which start the column equivalence process are Orbits 2 and 61 at the 14-th step, which means that column equivalence selects 11 nodes, which are 1, 7, 22, 23, 31, 47, 48, 54, 69, 70, and 78 (see Table 1 for the corresponding brain regions). Similarly, Supplementary Table 4 (*t* = 0.1) shows that Orbits 55 and 61 provide the first column equivalence. This occurs at the 19-th step, with the following six nodes involved: 1, 22, 31, 48, 69, and 78. Finally, from Figure 2 we select, at the 15-th step, Orbits 2 and 33 as the orbits which firstly show column equivalence. In this case, the set of the emerging 10 nodes is 1, 7, 22, 31, 47, 48, 54, 69, 70, and 78. Hence, whatever the threshold value, we recover the same persistent set of six nodes which are those above discussed in detail (see Table 2).

#### Randomization

To quantify whether our results differ from those obtained with randomly generated data, we have repeated the analysis by randomizing the 133 functional connectivity matrices with *degree preserving randomization* (DPR). DPR yields randomized matrices that share the same number of nodes, and the same degree of each node, with the original functional matrices. Note that, by construction, the network’s degree distribution does not change while, with a sufficient number of link rearrangements (or rewirings), the topological structure of the network becomes completely different.

**FIGURE 3:**
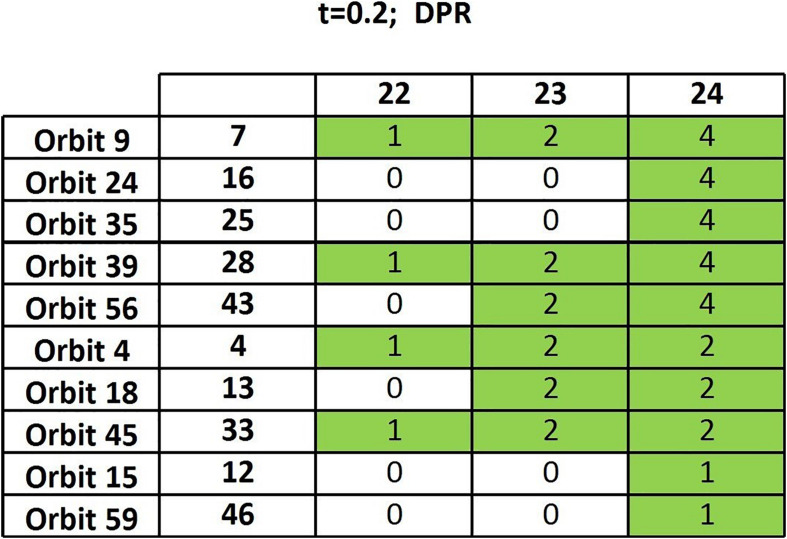
Output table of the algorithm of section “Frequency Table and Node Table” when randomized matrices are considered.

We have generated 133 DPR matrices from the thresholded functional connectivity matrices (*t* = 0.2) and run the algorithm of section “Frequency Table and Node Table” on such a random sample. The resulting output table (Figure 3) has a completely different structure compared to the one based on actual data (Figure 2): Only a 10 × 3 sub-table contains nonzero entries, showing very few persisting orbits and only in the very final steps. We can therefore safely conclude that the above-discussed results are meaningful as they are very far from those yielded by random data.

### GCM Comparison

**FIGURE 4:**
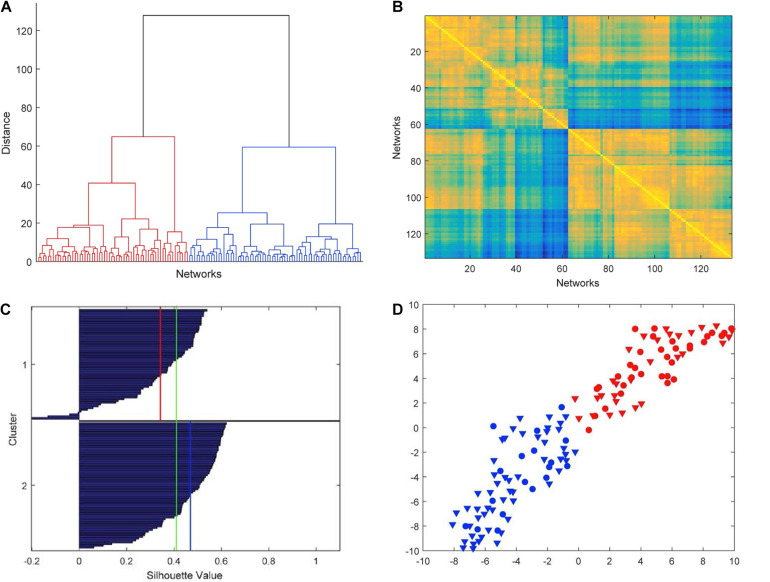
Results of the hierarchical cluster analysis based on five-node GCD. **(A)** The dendrogram obtained with Ward’s distance: the adopted two-cluster partition is evidenced by red/blue color. **(B)** Heatmap of the GCD matrix: “hot” colors represent short distances (high similarity) and vice versa “cold” colors. The order of rows/columns is consistent with the ordering of the leaves in the dendrogram. **(C)** Silhouette diagram: The red/blue lines mark the mean silhouette values of the respective clusters, and the green line the overall mean value. **(D)** t-SNE (stochastic neighbor embedding): In this 2D plot, the clusters are contiguous but well separated.

The hierarchical cluster analysis based on five-node GCD (threshold value *t* = 0.2) is summarized in the dendrogram of Figure 4A. We clearly note that the 133 networks are separated into two main clusters, which we denote by C1 (left in the picture, red, 62 networks) and C2 (right, blue, 71 networks). The two main clusters, in turn, can be partitioned into fairly well-defined subclusters, at least two in C2 and two or three in C1. In Figure 4B, the GCD matrix is visualized in the form of a heatmap, where the “hot” colors represent short distances (high similarity) and vice versa the “cold” colors. A “hot” block on the diagonal, if associated with “cold” areas outside the block, may reveal a significant cluster. The distinction between clusters C1 and C2 (the two main blocks on the diagonal) emerges quite clearly, whereas a finer partition is not so evident. Thus, from now on, we will only consider the above-described two-cluster partition.

The cluster analysis needs to be validated by assessing the significance of the obtained results. Figure 4C shows the silhouette plot (Kaufman and Rousseeuw, 1990) associated with the partition. In the plot, the silhouette value *s_i* of each network *i* (i.e., each object to be clustered) is displayed with a bar, and bars are grouped by cluster and organized in decreasing order. The silhouette value *s_i* is defined as

$$s_{i}=\frac{b_{i}-a_{i}}{\text{max}\,\left(a_{i},b_{i}\right)}$$

where *a_i* is the average distance from *i*to the other points of the same cluster, and *b_i* is the minimum (over clusters) of the average distances from *i* to the points in a different cluster. Thus, *s_i* ranges from −1 to +1, with a large value denoting that *i* is well-matched to the points in its own cluster. Typically, a large average silhouette value over a cluster reveals its significance, and a large average value over all objects *i* denotes a meaningful partition. In our case, the averages are 0.34 and 0.47 for C1 and C2, respectively, and 0.41 for the entire pool. These values are sufficiently large to denote a fair quality of the partition. Additionally, while a large number of negative $s_{i}^{\prime }$s is a typical indicator of an incorrect partitioning, in our case we only have a very limited number of them (4 out of 133, see Figure 4C).

Further evidence of the validity of the results of the cluster analysis is provided by t-distributed stochastic neighbor embedding (t-SNE) (van der Maaten and Hinton, 2008), an algorithm aimed at embedding high-dimensional points in a low-dimensional space trying to reproduce the similarities between points by matching their distance. Although it is impossible, in general, to exactly match distances in a low-dimensional projection, the algorithm—which is based on matching suitable probabilistic descriptions of the point sets in the high- and low-dimensional spaces—is typically successful in visualizing the original clusters, if any, even in the low-dimensional projection. By using the Matlab implementation provided by the authors of van der Maaten and Hinton (2008)^1^, we have obtained the t-SNE plot of Figure 4D, which confirms that our two clusters C1 and C2 are well separated although contiguous.

We now move to investigate the possible relationship between the above-described two-cluster partition and the available attributes (sex and age) of the subjects. Table 3 compares the percentages of males and females of the entire sample with that of the two clusters. In bold, we highlight the values that are larger than those of the entire sample (shown in the last row).

**TABLE 3:** Percentage of males and females in clusters C1 and C2.

	# Networks	% Males	% Females
**Cluster C1**	62	**53.2%**	46.8%
**Cluster C2**	71	25.3%	**74.7%**
**Total**	133	38.3%	61.7%

*The values larger than the corresponding values in the whole sample are in bold. Cluster C1 (resp., C2) has a significant predominance of male (resp., female) subjects with respect to the whole sample (p = 0.012, binomial test; p = 0.001, permutation test with 1,000 permutations).*

At first glance, it results that C1 tends to aggregate more males than the whole sample, while C2 aggregates more females. This result is statistically significant (*p* = 0.012, binomial test; *p* = 0.001, permutation test with 1,000 permutations), so it is possible to claim that the structure of the networks—evaluated in terms of GDVs—is different between males and females, at least limited to this sample.

In order to explore the role of age, we have arranged the 133 subjects in seven groups of males and seven groups of females having similar age, as shown in Table 4.

**TABLE 4:** Subgroups of subjects, with the corresponding age ranges and cardinalities.

Subgroup	Age range	# Males	# Females
g1	6–15	10	9
g2	16–25	7	18
g3	26–35	9	11
g4	36–45	7	9
g5	46–57	6	13
g6	58–70	5	10
g7	71–79	7	12

If we turn our attention toward the age classes, the statistical analysis does not find a significant relationship between clusters and age groups. As a matter of fact, if we compare what we found in the age groups in clusters C1 and C2 with the reference percentages in the whole sample, we do not observe any remarkable relationship between clusters and age groups. Indeed, the percentage highlighted in bold, and the reference values shown in the last row in Table 5 are really close.

**TABLE 5:** Percentage of subjects in the seven age groups for clusters C1 and C2.

	# networks	g1	g2	g3	g4	g5	g6	g7
**Cluster C1**	62	**16.1%**	16.1%	**16.1%**	**12.9%**	12.9%	**14.5%**	11.3%
**Cluster C2**	71	12.7%	**21.1%**	14.1%	11.3%	**15.5%**	8.4%	**16.9%**
**Total**	133	14.3%	18.8%	15.0%	12.0%	14.3%	11.3%	14.3%

*The bold values represent the larger percentage of subjects in the seven age groups for clusters C1 and C2.*

As shown in Table 6, the same negative evidence is also found by aggregating the classes g1, g2, and g3 in a single macro-class, called gA (which therefore includes subjects up to 35 years), and the rest in another macro-class, called gB. The relationship between age macro-class and cluster is very weak, if not null, and by no means statistically significant (*p* = 0.533, binomial test; *p* = 0.416, permutation test with 1,000 permutations).

**TABLE 6:** Percentage of subjects in the macro age class gA (subjects up to 35 years old) and gB (subject from 36 to 79 years old) in clusters C1 and C2.

	# networks	gA	gB
**Cluster C1**	62	**48.4%**	51.6%
**Cluster C2**	71	47.9%	**52.1%**
**Total**	133	48.1%	51.9%

*The values larger than the corresponding values in the whole sample are in bold. No evidence of a significant relationship between clusters and macro age class emerges (p = 0.533, binomial test; p = 0.416, permutation test with 1,000 permutations).*

## Concluding Remarks

We have implemented a graphlet-based topological algorithm for investigating the RS network. Due to its flexibility, it could be employed in other fields such as bioinformatics, genetics, and social interaction. The proposed algorithm proved able to yield a topological selection of the main areas of the DMN, as well as a number of related brain regions that are known in the literature to strongly interact with the DMN. Thus, we have obtained a topological characterization of the RS, which seems to be very promising in view of possible further neurobiological applications.

We have also compared the different performance of the subjects at RS by means of a topological distance between pairs of brain networks. The cluster analysis and the statistical analysis carried out downstream showed a rather clear separation of the 133 networks analyzed in two well-defined clusters: The sex of the subjects seems to play an important role in determining the distance between pairs of networks, differently from the age that, on the contrary, seems not to play. This preliminary result prompts future investigations on the predictive power of graphlets in brain network analysis.

As far as we know, graphlets have been applied mainly in bioinformatics and in economics. An example is their application in cellular network analysis, in particular in protein–protein interaction (PPI) networks (see for example Pascual et al., 2013). Of course, our proposal should be intended as a first step of a graphlet-based approach to the investigation of the brain network, and several other studies are needed to consolidate the promising topological outcomes that we have obtained, possibly extending the analysis to subjects affected by neurological or mental diseases. We believe that, in the near future, the graphlet approach could prove to be a powerful tool in diagnostic, enabling to support and reinforce the traditional clinical analysis.

We would like to conclude by giving two prospective future studies of potential impact that would be worth exploring by means of graphlets, namely, the investigation of negative entries in the fMRI matrices, and possible biological interpretation of graphlets.

The problem of a correct interpretation of the negative entries is undoubtedly of great relevance in neuroscience, since, as far as we know, their neurobiological meaning has not been fully understood yet, though some approaches have been proposed to throw light on this topic (see for example Chen et al., 2011; Zhan et al., 2017). In this view, and since the proposed algorithm runs independently of the nature of the input data, we tried to apply the same graphlet approach to the negative part of the fMRI matrices. Our guess was that some subset of the so-called “anti-DMN” (see for example Di Perri et al., 2016; Velichkovsky et al., 2018) would have been selected. Differently, no stable orbits in fact emerged, and consequently no subset of topologically relevant nodes could be clearly determined. At a first glance, the absence of an emerging anti-DMN was quite surprising, but, at a deeper analysis, we realized that this is perfectly consistent with the available dataset, since the exploited Atlas misses the main brain expected areas. As a consequence, it could be of interest to reproduce the proposed approach with a suitable setting.

Regarding the possible biological meaning of graphlets, we remark that graphlet-based measures are already considered and seem to provide promising and powerful methods in the investigation of biological network (see for example Pržulj, 2007; Hayes et al., 2013). In our manuscript, working at a macro scale, we have pointed out how specific orbits play different topological roles in the functional activity of the brain. There is no doubt that the investigation of possible biological interpretations of graphlets could be even explored at a microscale resolution, even if this would require a completely different parceling of the involved brain regions.

## Data Availability Statement

Publicly available datasets were analyzed in this study. This data can be found here: https://web.gin.g-node.org (doi: 10.12751/g-node.ef14cc).

## Ethics Statement

The studies involving human participants were reviewed and approved by the Ethics Committee of the Don Gnocchi Foundation. Written informed consent to participate in this study was provided by the participants’ legal guardian/next of kin.

## Author Contributions

PF and PD: study conception and design. PF, CP, and EM: analysis of results. PF, PD, and CP: interpretation of results and draft manuscript preparation. All authors reviewed the results and approved the final version of the manuscript.

## Conflict of Interest

The authors declare that the research was conducted in the absence of any commercial or financial relationships that could be construed as a potential conflict of interest.

## Footnotes

1 https://lvdmaaten.github.io/tsne/
