# Supplementary material for: A Graphlet-Based Topological Characterization of the Resting-State Network in Healthy People
Source: Front Neurosci. 2021 Apr 28;15:665544. doi: 10.3389/fnins.2021.665544 (PMC8113409; doi:10.3389/fnins.2021.665544)
Supplement: Supplementary file 1 [file Data_Sheet_1.PDF]

Supplementary Material for "A Graphlet-based Topological Characterization of the Resting State Network in Healthy People"

Paolo Finotelli[1*], Carlo Piccardi[2], Edie Miglio[1], Paolo Dulio[1]

[1] Department of Mathematics, Politecnico di Milano, Italy

[2] Department of Electronics, Information and Bioengineering, Politecnico di Milano, Italy

Table 1SM: The sorted frequency table, whose entries are called global orbit touching frequencies. The nodes characterized by such frequencies are shown in Table 2SM.

Orbit 0	Orbit 1	Orbit 2	Orbit 4	Orbit 6	Orbit 8	Orbit 9	Orbit 10	Orbit 11	Orbit 12
F	F	F	F	F	F	F	F	F	F
114	56	117	58	66	55	60	73	119	55
110	51	110	53	59	54	58	69	111	51
104	49	104	53	57	54	56	68	104	51
103	47	102	50	57	53	54	64	103	50
97	47	97	50	56	53	54	63	97	49
90	45	85	49	55	53	54	62	86	49
82	45	79	49	55	51	54	62	79	49
79	45	78	48	53	49	53	61	79	49
76	45	78	48	52	47	52	61	78	46
75	45	77	48	51	47	52	60	76	45
75	44	75	47	51	47	52	60	74	45
63	44	69	47	50	46	51	60	65	45
62	44	65	46	50	46	51	60	63	45
60	44	64	46	50	46	50	59	60	44
59	43	63	46	49	45	49	58	60	43
57	43	60	44	49	45	49	58	60	43
56	43	59	44	47	44	48	57	58	43
54	42	57	44	46	44	48	57	58	42
52	42	57	43	46	44	48	57	58	42
51	42	57	43	46	43	47	56	57	42
49	42	57	43	46	43	46	55	57	41
47	41	57	43	45	42	46	50	55	41
47	40	56	42	42	42	46	50	54	40
45	40	53	42	42	41	45	49	52	40

Orbit 13	Orbit 15	Orbit 18	Orbit 19	Orbit 22	Orbit 24	Orbit 25	Orbit 27	Orbit 29	Orbit 30
F	F	F	F	F	F	F	F	F	F
119	60	59	67	65	67	78	67	65	111
111	60	57	66	64	64	67	62	60	110
109	59	57	62	63	62	67	62	59	100
107	59	57	59	61	62	61	62	59	99
100	58	57	58	60	60	57	61	58	93
93	58	57	58	58	60	54	61	57	83
87	57	56	57	56	59	54	58	57	83
83	55	55	56	55	58	52	57	56	79
79	55	54	56	53	57	52	53	54	78
79	55	54	54	52	57	49	51	54	77
79	54	53	53	52	57	47	50	54	74
72	54	52	53	51	57	47	50	54	63
69	53	51	53	50	57	47	50	53	60
64	52	50	53	50	55	47	49	53	58
63	52	50	53	50	55	45	48	53	58
63	52	50	51	49	53	45	48	52	57
60	51	50	50	49	52	45	48	52	57
60	50	49	50	49	52	44	48	50	57
58	50	49	49	47	51	44	47	49	56
52	50	48	48	47	50	43	47	49	56
52	49	48	48	46	49	42	47	48	56
51	49	48	48	46	49	42	47	48	55
50	48	48	48	46	49	42	46	47	55
49	48	47	47	46	49	41	46	47	54

Orbit 31	Orbit 32	Orbit 33	Orbit 34	Orbit 35	Orbit 36	Orbit 37	Orbit 39	Orbit 40	Orbit 41
F	F	F	F	F	F	F	F	F	F
69	69	116	61	68	60	64	69	62	100
67	68	109	59	65	57	55	67	58	98
61	67	104	57	63	57	53	67	58	96
61	65	101	55	62	57	51	66	56	94
61	57	95	54	60	56	48	65	54	88
59	55	85	53	58	54	48	65	50	84
59	54	76	51	57	53	48	64	50	82
59	54	76	50	57	51	47	59	50	80
59	53	75	50	57	51	46	59	50	79
58	53	75	49	56	50	46	57	48	77
58	49	74	49	56	48	45	57	48	73
57	48	73	49	56	47	45	56	47	70
55	48	72	48	54	46	45	56	47	69
52	48	62	45	54	44	45	55	47	65
51	48	60	45	53	43	45	55	46	63
51	47	59	45	53	43	43	54	46	59
51	47	58	43	52	43	42	53	45	57
51	47	57	43	52	43	42	53	45	55
49	44	57	42	52	42	41	53	44	55
49	44	55	41	52	42	41	52	44	52
49	43	54	41	51	41	40	51	43	51
49	43	53	40	50	41	40	51	43	51
48	41	52	39	50	40	40	48	43	51
48	41	52	39	48	40	40	48	43	49

Orbit 42	Orbit 43	Orbit 45	Orbit 46	Orbit 48	Orbit 49	Orbit 50	Orbit 51	Orbit 52	Orbit 53
F	F	F	F	F	F	F	F	F	F
119	61	63	60	106	58	61	61	73	81
109	60	59	59	102	54	60	58	57	78
105	56	59	58	101	54	59	56	57	77
104	54	58	58	99	49	58	55	56	75
97	54	56	54	92	48	57	55	55	73
84	52	56	54	91	47	56	53	55	73
82	51	55	53	86	47	53	51	52	70
77	51	55	52	84	47	51	49	52	66
77	50	55	52	80	46	51	48	52	64
77	49	54	52	80	45	50	47	51	61
75	47	54	51	76	45	49	47	50	60
68	46	54	50	73	45	49	45	50	59
65	46	54	48	72	44	49	45	50	55
65	45	52	47	67	44	49	43	48	55
63	45	52	47	66	43	49	43	48	53
60	45	51	47	65	42	49	43	47	52
59	45	50	46	62	42	49	43	47	51
58	45	50	46	61	42	48	42	46	51
58	44	49	46	54	42	46	41	45	51
57	44	49	44	53	42	46	41	44	51
55	44	49	41	51	41	45	41	44	50
55	43	48	41	50	41	44	41	42	50
55	42	46	41	50	41	44	41	42	48
54	41	46	40	49	41	43	40	42	48

Orbit 54	Orbit 55	Orbit 56	Orbit 57	Orbit 58	Orbit 59	Orbit 60	Orbit 61	Orbit 62	Orbit 63
F	F	F	F	F	F	F	F	F	F
57	116	66	86	115	56	71	121	59	61
57	112	64	85	109	53	69	111	54	59
56	107	64	80	103	52	66	106	53	55
54	104	61	79	103	50	65	106	51	54
53	98	61	78	97	49	64	97	49	54
50	98	59	77	86	49	63	89	48	53
50	89	58	76	80	49	61	83	47	53
50	82	57	74	74	48	60	81	46	52
49	82	57	74	74	47	59	81	46	50
49	81	56	73	73	45	57	77	46	49
49	78	55	73	73	44	57	76	45	49
49	70	54	71	66	44	57	69	45	48
49	69	54	66	61	44	57	64	44	48
48	64	54	66	60	43	57	64	43	48
48	63	53	63	60	43	53	61	43	48
47	63	52	62	59	43	50	58	42	47
46	63	52	62	59	43	50	58	42	45
46	60	51	62	58	43	50	58	42	44
45	59	51	61	56	43	49	56	42	44
44	55	51	61	54	42	49	55	42	44
43	55	50	60	53	42	49	54	41	44
43	53	49	59	52	41	48	54	41	44
42	53	48	55	51	41	48	53	41	43
42	53	47	52	49	40	47	50	40	42

Orbit 64	Orbit 65	Orbit 66	Orbit 67	Orbit 68	Orbit 70
F	F	F	F	F	F
87	57	72	119	57	61
82	53	67	109	53	57
80	51	66	108	52	56
79	50	65	107	51	55
77	49	65	98	51	51
76	48	59	94	50	50
75	48	59	87	49	49
71	47	58	84	49	46
69	46	57	82	49	46
68	46	57	81	48	46
65	45	57	80	48	45
64	45	56	71	48	45
63	44	56	70	47	45
62	44	56	63	47	45
61	44	55	63	46	44
59	43	55	62	45	43
58	43	54	60	44	42
57	43	54	60	43	42
57	42	52	57	43	42
55	42	51	51	43	42
50	42	51	51	43	42
50	41	51	51	43	42
49	41	49	50	43	41
48	41	49	48	43	41

Table 2SM: The sorted node table. The corresponding global orbit touching frequencies are shown in Table 1SM. The DMN nodes are illustrated in black bold font.

Orbit 0	Orbit 1	Orbit 2	Orbit 4	Orbit 6	Orbit 8	Orbit 9	Orbit 10	Orbit 11	Orbit 12
N	N	N	N	N	N	N	N	N	N
**48**	63	**48**	51	56	8	11	47	**48**	32
**1**	56	**1**	4	38	55	58	71	**1**	24
22	16	22	66	11	63	**25**	**31**	22	79
69	13	69	**20**	16	33	35	40	69	**21**
**31**	**25**	**31**	91	9	49	51	32	**31**	13
**78**	6	**78**	3	**25**	60	56	70	**78**	60
70	**10**	54	58	58	**10**	85	94	54	63
54	**21**	47	2	18	**1**	38	17	70	66
47	32	70	11	63	2	9	79	47	28
7	33	7	19	5	13	16	24	7	**10**
23	9	23	34	52	**48**	27	36	23	65
36	18	55	92	32	29	5	**77**	36	86
83	60	8	5	51	42	81	**78**	83	90
64	80	83	6	90	**57**	41	69	8	18
17	58	36	45	13	76	34	**30**	17	**67**
**77**	**67**	94	16	85	80	63	87	55	**68**
**30**	71	17	38	81	**21**	6	22	**57**	71
94	4	**30**	73	6	24	15	64	64	4
76	38	60	**25**	26	**78**	44	83	**77**	8
**57**	50	64	50	35	6	**72**	7	**30**	**77**
55	52	76	53	45	**12**	**20**	23	94	33
8	24	**77**	63	39	75	91	**1**	76	**72**
**10**	51	**57**	49	27	86	92	**48**	60	6
60	**57**	**10**	**67**	41	28	53	54	**10**	**12**

Orbit 13	Orbit 15	Orbit 18	Orbit 19	Orbit 22	Orbit 24	Orbit 25	Orbit 27	Orbit 29	Orbit 30
**N**	**N**	**N**	**N**	**N**	**N**	**N**	**N**	**N**	**N**
**48**	44	45	58	56	11	32	27	17	**48**
**1**	45	19	38	38	85	71	34	**77**	**1**
22	34	27	11	11	15	79	44	**30**	22
69	51	34	56	16	35	24	91	83	69
**31**	11	51	5	58	34	18	74	75	**31**
**78**	92	91	35	9	81	40	81	**67**	70
70	91	66	9	52	38	65	11	87	**78**
47	27	74	16	81	44	**30**	45	36	47
7	74	44	85	85	5	**68**	35	28	7
23	81	92	90	**25**	45	39	92	32	54
54	19	88	15	35	58	13	2	64	23
83	35	5	**25**	34	62	47	3	79	83
36	73	81	45	45	91	**77**	**20**	40	55
17	5	3	52	51	82	94	62	65	**10**
64	62	50	88	90	92	36	15	**68**	60
76	88	58	51	5	56	46	19	4	36
30	**20**	62	81	13	9	63	38	66	64
**77**	58	11	92	63	26	**10**	88	23	94
94	66	20	34	15	16	86	41	7	17
**10**	82	15	27	88	41	60	50	24	**30**
**57**	15	35	41	18	27	**21**	66	**21**	**57**
60	85	41	63	26	51	**57**	73	70	8
55	3	73	82	41	53	87	**25**	18	**77**
75	38	26	26	82	88	4	80	94	75

Orbit 31	Orbit 32	Orbit 33	Orbit 34	Orbit 35	Orbit 36	Orbit 37	Orbit 39	Orbit 40	Orbit 41
**N**	**N**	**N**	**N**	**N**	**N**	**N**	**N**	**N**	**N**
38	79	**48**	63	38	67	63	38	32	**1**
11	24	**1**	8	11	2	13	34	18	22
56	32	22	55	58	49	33	58	24	69
58	71	69	49	35	55	42	11	63	**48**
81	**68**	**31**	**67**	85	66	**10**	81	**79**	**31**
9	**30**	78	33	34	8	60	85	52	**78**
15	65	54	**12**	5	42	80	15	56	47
16	94	55	2	51	63	55	56	**68**	54
34	18	23	13	88	80	8	91	71	23
35	**77**	70	42	15	33	24	5	13	70
85	36	47	60	41	**59**	6	35	**25**	7
5	7	7	80	82	76	29	16	4	17
**25**	40	8	**10**	44	6	52	41	65	83
91	83	83	**59**	45	**21**	56	9	90	36
41	87	36	66	62	19	**57**	82	5	64
44	4	**57**	76	92	**25**	49	88	16	94
51	13	60	**25**	16	29	2	**25**	39	**30**
88	47	**30**	28	**25**	93	39	44	66	76
13	**20**	94	6	81	60	71	51	6	**77**
52	70	17	16	91	75	89	92	**10**	**57**
82	42	**77**	**68**	26	4	9	27	**21**	**10**
92	86	**10**	5	**20**	**12**	16	62	51	40
27	17	33	57	27	3	**21**	14	**72**	87
63	28	76	**72**	52	89	46	45	86	28

Orbit 42	Orbit 43	Orbit 45	Orbit 46	Orbit 48	Orbit 49	Orbit 50	Orbit 51	Orbit 52	Orbit 53
**N**	**N**	**N**	**N**	**N**	**N**	**N**	**N**	**N**	**N**
**48**	28	91	67	**48**	63	**48**	8	32	**1**
**1**	**25**	11	66	**1**	13	55	2	24	69
69	**67**	15	4	69	56	**1**	55	79	**48**
22	8	34	**21**	22	**10**	8	33	87	22
**31**	51	45	32	**78**	42	33	49	65	**31**
**78**	79	92	51	**31**	6	**10**	63	71	70
70	**72**	35	65	70	9	60	**59**	18	47
7	75	44	19	47	52	63	**67**	28	**78**
47	4	88	**20**	7	32	69	80	75	7
54	**12**	27	24	54	5	70	13	**68**	23
23	**68**	38	18	23	18	7	42	**30**	55
55	**21**	62	**68**	83	55	22	6	63	54
8	**77**	74	53	64	33	29	**25**	66	**57**
36	**20**	58	43	**30**	80	49	3	**67**	94
83	36	85	86	36	16	**57**	52	86	**10**
17	55	82	90	17	2	76	60	4	36
**57**	**59**	**20**	73	**77**	24	**78**	**72**	**77**	8
**77**	83	81	79	94	**25**	**31**	76	**12**	29
94	2	5	87	75	39	**12**	5	17	64
64	76	**25**	6	76	81	54	**10**	40	83
**30**	94	41	42	40	8	75	**12**	51	**30**
60	17	51	71	29	38	13	81	6	60
76	24	14	89	**57**	49	80	90	26	17
**10**	**30**	19	39	**10**	90	17	16	36	75

Orbit 54	Orbit 55	Orbit 56	Orbit 57	Orbit 58	Orbit 59	Orbit 60	Orbit 61	Orbit 62	Orbit 63
**N**	**N**	**N**	**N**	**N**	**N**	**N**	**N**	**N**	**N**
32	**48**	15	47	**48**	51	**1**	**48**	8	**1**
63	**1**	34	69	**1**	66	22	**1**	2	55
18	69	81	**31**	22	63	69	22	49	**57**
56	22	11	23	69	**25**	**48**	69	33	8
79	**31**	85	22	**31**	4	**78**	**31**	13	**10**
4	**78**	91	70	**78**	6	70	**78**	**59**	33
16	70	44	**78**	54	**67**	47	54	6	63
39	47	58	**30**	7	**21**	**31**	47	55	**48**
5	54	62	64	47	32	23	70	63	49
13	23	35	7	23	**68**	7	7	**67**	13
24	7	5	17	70	18	54	23	80	60
**25**	36	16	**48**	83	24	55	83	81	**21**
58	83	45	54	8	58	**57**	36	**10**	29
6	**30**	82	**77**	17	13	64	64	9	69
51	17	56	32	36	16	**10**	17	52	**78**
90	64	9	36	**30**	19	8	**30**	5	76
38	**77**	38	79	**57**	43	**12**	76	16	7
**72**	94	27	94	55	50	60	**77**	**25**	17
26	76	88	40	64	90	28	60	41	22
52	**10**	92	71	94	**20**	**77**	55	76	24
9	55	**25**	**1**	75	53	83	**10**	42	70
66	8	26	83	60	56	**30**	94	56	75
**10**	**57**	14	87	**77**	**72**	36	**57**	66	80
93	60	74	24	76	8	17	8	20	**12**

Orbit 64	Orbit 65	Orbit 66	Orbit 67	Orbit 68	Orbit 70
**N**	**N**	**N**	**N**	**N**	**N**
47	51	71	**48**	8	24
**31**	63	47	**1**	33	79
70	**25**	32	22	**10**	**21**
**78**	6	24	69	60	32
69	11	79	**31**	75	60
23	16	36	**78**	**21**	86
22	58	87	70	**12**	63
**1**	38	17	47	24	13
**48**	4	23	54	29	65
64	56	**31**	23	13	**68**
36	9	64	7	**57**	18
7	**20**	**30**	83	**78**	**67**
83	**21**	70	36	**1**	71
94	66	94	17	55	87
17	**67**	40	**77**	22	90
54	52	**78**	64	**48**	28
40	53	22	**30**	**30**	4
**30**	81	**77**	76	7	**10**
**77**	18	7	94	42	42
87	**72**	65	55	**68**	**57**
**57**	85	69	**57**	69	66
75	8	83	60	70	**77**
71	**12**	29	**10**	76	6
76	90	**68**	75	80	43

Table 3SM: Output of the analysis carried out by considering F^+.

t=0.0

	steps										
	**14**	**15**	**16**	**17**	**18**	**19**	**20**	**21**	**22**	**23**	**24**
**Orbit 0**	0	0	0	0	2	7	8	8	8	11	12
**Orbit 2**	1	1	1	1	4	7	8	8	8	11	12
**Orbit 11**	0	0	0	0	4	7	8	8	8	11	12
**Orbit 13**	0	0	0	1	2	7	8	8	8	11	12
**Orbit 30**	0	0	0	0	0	0	8	8	8	11	12
**Orbit 33**	0	0	0	0	4	7	8	8	8	11	12
**Orbit 42**	0	0	0	0	1	1	1	2	2	11	12
**Orbit 48**	0	0	0	0	0	0	0	2	2	11	12
**Orbit 50**	0	0	0	0	0	0	0	0	0	0	12
**Orbit 55**	0	0	0	0	1	1	1	2	2	11	12
**Orbit 58**	0	0	0	0	4	7	8	8	8	11	12
**Orbit 61**	1	1	1	1	4	7	8	8	8	11	12
**Orbit 67**	0	0	0	1	2	7	8	8	8	11	12
**Orbit 12**	0	0	0	0	0	0	0	0	1	1	4
**Orbit 25**	0	0	0	0	0	0	0	0	0	0	4
**Orbit 40**	0	0	0	0	0	0	0	0	0	0	4
**Orbit 52**	0	0	0	0	0	0	0	0	1	1	4
**Orbit 54**	0	0	0	0	0	0	0	0	0	0	4
**Orbit 1**	0	0	0	0	0	0	0	0	0	0	3
**Orbit 8**	0	0	0	0	0	0	0	0	0	0	3
**Orbit 34**	0	0	0	0	0	0	0	0	0	0	3
**Orbit 37**	0	0	0	0	0	0	0	0	0	1	3
**Orbit 41**	0	0	0	0	0	0	0	1	1	1	3
**Orbit 49**	0	0	0	0	0	0	0	0	0	1	3
**Orbit 51**	0	0	0	0	0	0	0	0	0	1	3
**Orbit 53**	0	0	0	0	0	0	0	0	0	0	3
**Orbit 60**	0	0	0	0	0	0	0	1	1	1	3
**Orbit 62**	0	0	0	0	0	0	0	0	0	1	3
**Orbit 63**	0	0	0	0	0	0	0	0	0	0	3
**Orbit 68**	0	0	0	0	0	0	0	0	0	0	3
**Orbit 4**	0	0	0	0	0	0	0	0	0	0	2
**Orbit 10**	0	0	0	0	0	0	0	0	0	0	2
**Orbit 31**	0	0	0	0	0	0	0	0	0	1	2
**Orbit 35**	0	0	0	0	0	0	0	0	0	1	2
**Orbit 39**	0	0	0	0	0	0	0	0	0	0	2
**Orbit 57**	0	0	0	0	0	0	0	0	0	0	2
**Orbit 59**	0	0	0	0	0	0	0	0	0	0	2
**Orbit 64**	0	0	0	0	0	0	0	0	0	0	2
**Orbit 65**	0	0	0	0	0	0	0	0	0	0	2
**Orbit 6**	0	0	0	0	0	0	0	1	1	1	1
**Orbit 9**	0	0	0	0	0	0	0	0	0	0	1
**Orbit 22**	0	0	0	0	0	0	0	1	1	1	1
**Orbit 24**	0	0	0	0	0	0	0	0	0	0	1
**Orbit 36**	0	0	0	0	0	0	0	0	0	0	1
**Orbit 46**	0	0	0	0	0	0	0	0	0	0	1

Table 4SM: Output of the analysis carried out by considering F^{+} thresholded at t=0.1.

t=0.1

	step					
	19	20	21	22	23	24
Orbit 4	0	0	0	0	0	13
Orbit 6	0	0	0	0	2	13
Orbit 9	0	0	0	0	8	13
Orbit 15	0	1	3	4	8	13
Orbit 18	0	0	0	4	8	13
Orbit 19	0	0	3	4	8	13
Orbit 22	0	0	0	1	2	13
Orbit 24	0	1	3	4	8	13
Orbit 27	0	1	2	2	8	13
Orbit 31	0	0	3	4	8	13
Orbit 35	0	0	0	1	2	13
Orbit 39	0	0	2	2	8	13
Orbit 45	0	1	2	2	8	13
Orbit 62	0	0	0	0	0	13
Orbit 2	0	1	2	4	7	8
Orbit 11	0	0	0	1	7	8
Orbit 30	0	0	0	0	0	8
Orbit 33	0	0	2	4	7	8
Orbit 42	0	1	2	4	7	8
Orbit 55	1	1	1	4	7	8
Orbit 58	0	0	0	1	7	8
Orbit 61	1	1	1	4	7	8
Orbit 67	0	0	0	0	7	8
Orbit 12	0	0	0	0	0	4
Orbit 37	0	0	0	0	0	4
Orbit 40	0	0	0	0	0	4
Orbit 49	0	0	0	0	0	4
Orbit 54	0	0	0	0	0	4
Orbit 1	0	0	0	0	3	3
Orbit 34	0	0	0	1	3	3
Orbit 36	0	0	0	0	3	3
Orbit 51	0	0	0	1	3	3
Orbit 50	0	0	0	0	0	2
Orbit 53	0	0	0	0	0	2
Orbit 60	0	0	0	0	0	2
Orbit 0	0	0	0	0	1	1
Orbit 10	0	0	1	1	1	1
Orbit 13	0	0	0	0	1	1
Orbit 25	0	0	0	0	0	1
Orbit 29	0	0	0	0	0	1
Orbit 32	0	0	0	0	0	1
Orbit 52	0	0	0	0	0	1
Orbit 59	0	0	0	0	0	1
Orbit 63	0	0	0	0	0	1
Orbit 65	0	0	0	0	0	1
Orbit 66	0	0	1	1	1	1
Orbit 70	0	0	0	0	0	1
